# Altered Gray-White Matter Boundary Contrast in Toddlers at Risk for Autism Relates to Later Diagnosis of Autism Spectrum Disorder

**DOI:** 10.3389/fnins.2021.669194

**Published:** 2021-06-17

**Authors:** Michel Godel, Derek S. Andrews, David G. Amaral, Sally Ozonoff, Gregory S. Young, Joshua K. Lee, Christine Wu Nordahl, Marie Schaer

**Affiliations:** ^1^Department of Psychiatry, University of Geneva School of Medicine, Geneva, Switzerland; ^2^Department of Psychiatry and Behavioral Sciences, The Medical Investigation of Neurodevelopmental Disorders (MIND) Institute, UC Davis School of Medicine, University of California, Davis, Sacramento, CA, United States

**Keywords:** autism spectrum disorder, sibling risk, toddlers, neurodevelopment, neuroimaging, FreeSurfer

## Abstract

**Background:**

Recent neuroimaging studies have highlighted differences in cerebral maturation in individuals with autism spectrum disorder (ASD) in comparison to typical development. For instance, the contrast of the gray-white matter boundary is decreased in adults with ASD. To determine how gray-white matter boundary integrity relates to early ASD phenotypes, we used a regional structural MRI index of gray-white matter contrast (GWC) on a sample of toddlers with a hereditary high risk for ASD.

**Materials and Methods:**

We used a surface-based approach to compute vertex-wise GWC in a longitudinal cohort of toddlers at high-risk for ASD imaged twice between 12 and 24 months (*n* = 20). A full clinical assessment of ASD-related symptoms was performed in conjunction with imaging and again at 3 years of age for diagnostic outcome. Three outcome groups were defined (ASD, *n* = 9; typical development, *n* = 8; non-typical development, *n* = 3).

**Results:**

ASD diagnostic outcome at age 3 was associated with widespread increases in GWC between age 12 and 24 months. Many cortical regions were affected, including regions implicated in social processing and language acquisition. In parallel, we found that early onset of ASD symptoms (i.e., prior to 18-months) was specifically associated with slower GWC rates of change during the second year of life. These alterations were found in areas mainly belonging to the central executive network.

**Limitations:**

Our study is the first to measure maturational changes in GWC in toddlers who developed autism, but given the limited size of our sample results should be considered exploratory and warrant further replication in independent and larger samples.

**Conclusion:**

These preliminary results suggest that ASD is linked to early alterations of the gray-white matter boundary in widespread brain regions. Early onset of ASD diagnosis constitutes an independent clinical parameter associated with a specific corresponding neurobiological developmental trajectory. Altered neural migration and/or altered myelination processes potentially explain these findings.

## Background

Autism Spectrum Disorder (ASD) is a heterogeneous neurodevelopmental disorder characterized by difficulties in the domains of social interactions and communication, along with repetitive behaviors and restricted interests (American Psychiatric Association, 2013; Lai et al., 2014). ASD affects 1 in 54 individuals with an increasing prevalence over the past decades (Weintraub, 2011; Maenner et al., 2020). Etiological mechanisms of ASD are thought to be mainly due to complex interactions of genetic predisposition and environmental risk factors, but have not been fully elucidated (Huguet et al., 2013). It is now established that early intensive specific intervention can result in lasting positive outcomes (Reichow, 2012). Despite recent improvement in symptom screening tools and procedures, often the age of ASD diagnoses still remains too late to capitalize on a critical therapeutic window for intervention (Daniels et al., 2014; Brett et al., 2016). Even when specific intervention is delivered early, clinical response is highly variable between toddlers for reasons that are not yet fully explained (Howlin et al., 2009). Urgency for earlier diagnosis, intervention and more targeted therapeutic recommendations have led researchers to explore early behavioral and neurobiological markers of ASD.

Siblings of individuals with ASD share common genetic variants and exhibit an estimated risk of 18% to develop the disorder (Ozonoff et al., 2011). Studies on these children at high risk for ASD (HR) have allowed a better characterization of early clinical signs and trajectories of ASD (Szatmari et al., 2016). For example, we now know that the first reliable signs of ASD usually emerge during the second year of life (Sacrey et al., 2018) and are often preceded by less specific atypical behaviors in infancy (Sacrey et al., 2020). At the age of 18 months, approximately one-third of children who ultimately receive an ASD diagnosis get a stable and reliable diagnosis after a standardized assessment, while the other two-third will not yet demonstrate the full clinical presentation at this age (Ozonoff et al., 2015). This reduced clinical sensitivity of the early ASD behavioral phenotype has motivated the exploration of neuroimaging endophenotypes that could precede the emergence of symptoms and thus support clinical investigations (Wolff et al., 2018) as well as earlier identification of risk. Several magnetic resonance imaging (MRI) studies have found that children aged from 6 to 24 months who will later have a diagnosis of ASD exhibited a larger volume of extra-axial cerebrospinal fluid compared to typically developing children (TD) (Shen et al., 2013, 2017). Faster cortical surface expansion in infancy followed by brain volume overgrowth during the second year of life has also been shown to be predictive of later ASD diagnosis (The IBIS Network et al., 2017a) as have higher fractional anisotropy values at the age of 6 months and decreased values after 12 months in various deep white matter tracts such as the corpus callosum and inferior longitudinal fasciculus (Wolff et al., 2012, 2015). Despite these promising results, the imaging literature exploring early brain developmental signatures of later ASD diagnosis remains sparse. Furthermore, all the mentioned studies have focused on global morphological metrics, such as extra-axial cerebrospinal fluid volume or mean fractional anisotropy of major fiber tracts. To our knowledge, no studies have explored regional developmental differences in this population through either vertex-wise or voxel-wise methods to date.

In adults with ASD, alterations of the boundary between cerebral gray and white matter in widespread cortical areas have been identified using various methodological approaches. In histological studies less clear delineation of the transition between gray and white structures has been described in postmortem tissue of adult patients with ASD (Avino and Hutsler, 2010). *In vivo* assessment of the gray-white matter boundary has been conducted using an MRI morphometric based measure of gray-white matter intensity contrast (GWC) (Salat et al., 2009). GWC was first introduced in neurodegenerative imaging studies and has been extensively explored in aging populations (Alzheimer’s Disease Neuroimaging Initiative et al., 2015). In neurodevelopmental studies, GWC has been found decreased in many regions in school-aged children and adults with ASD, (Andrews et al., 2017) whereas adolescents with ASD have been found to exhibit similar GWC values compared to their TD peers (Andrews et al., 2017; MRC Aims Consortium et al., 2018). A more recent study reported increased GWC in adults with ASD, mostly in primary cortices (Fouquet et al., 2019). Although still sparse and somewhat inconsistent, this existing literature suggests that GWC is likely decreased in many secondary cortices amongst children and adults with ASD and increased in some primary cortical regions.

The precise biological mechanisms underlying these alterations is unknown. GWC alterations in autism have largely been attributed to neural migration deficits. This interpretation is supported by findings of abnormal migration of neurons in ASD (Packer, 2016). Identification of alterations in GWC very early in life would support this hypothesis, but to date, there have been no studies evaluating GWC in very young infants or toddlers with later ASD outcomes. To our knowledge, only one study in young TD toddlers reported increased GWC rates of change in areas relevant for language development between 12 and 19 months (e.g., the left superior temporal sulcus) (Travis et al., 2014) and GWC trajectories during the first years of life have never been assessed in ASD.

In the current study, we performed exploratory quantitative whole-brain surface-based analyses to test for associations between early GWC values and various ASD-related clinical parameters in a longitudinal cohort of HR infants to evaluate the potential of GWC as an early biomarker of diagnostic outcome and symptom severity. Each participant underwent two MRI scans between the age of 12 and 24 months. Given the lack of previous studies using GWC in children with ASD younger than 2, we didn’t have any *a priori* hypotheses regarding the location and direction of potential alterations. We assessed whether GWC was correlated with symptom severity at the time of the scan acquisition, and whether this was predictive of clinical diagnostic outcome at 36 months of age. Given the heterogeneity of age at which a stable and reliable diagnosis of ASD can be established (Ozonoff et al., 2015), we performed further *post hoc* exploratory analyses to evaluate the association between the age of first reliable diagnosis and GWC alterations. We hypothesized that if GWC alterations were to be found, they would be more prominent amongst ASD children with an early onset of ASD diagnosis (EOA) compared to children with a later onset of ASD diagnosis (LOA).

## Materials and Methods

We leveraged a MRI dataset involving participants recruited through the UC Davis MIND Institute between 2009 and 2011. The recruitment process as well as clinical and imaging procedures have been described in detail previously (Shen et al., 2013).

### Participants

Between 2009 and 2011, participants from a clinical longitudinal cohort (Ozonoff et al., 2010) were asked to also take part in an MRI acquisition protocol. Invitation was made through phone screening and led to the recruitment of 64 participants. For the current longitudinal analyses, we first selected the 41 participants who were categorized as HR (13 females). In this study, HR was defined as having an older sibling with a confirmed diagnosis of ASD. Having a sample exclusively constituted of HR participants allows to study the continuum of symptom severity and the emergence of ASD amongst participants who share a similar risk to develop the disorder (Ozonoff et al., 2011). For our analysis, we included only the HR participants who underwent 2 MRI scans (first at 12–15 months and second at 18–24 months of age). From the initial 41 HR participants, 6 were excluded (14.6% from initial sample) due to failure of one scan acquisition, 1 (2.4%) due to failure of both scan acquisitions, 4 (9.8%) for not coming to one MRI session, 7 (17.1%) due to dropping out from the study, 1 (2.4%) due to lost data, and 2 (4.9%) because of poor image quality (described below). From the overall 61 attempted scans, there were 9 failures resulting in a success rate of 85.2%. This resulted in a final sample of 20 HR children (5 females).

Demographic characteristics of the sample are displayed in Table 1. We divided our sample into three groups according to their diagnostic outcome at age 3: HR with typical development (HR-TD, *n* = 8, 3 females), HR with ASD (HR-ASD, *n* = 9, 1 female) and HR with atypical development (HR-non-TD, *n* = 3, 1 female). One HR-TD child did not undergo the 18-month clinical assessment but was not excluded from the study. As an additional exploratory analysis, we further separated HR-ASD participants into two subgroups according to age of first established diagnosis. HR-ASD who were diagnosed at 18 months or before were classified as early onset autism (EOA; *n* = 4, 1 female). HR-ASD participants whose diagnosis was established later than 18 months of age were labeled as later onset autism (LOA; *n* = 5, 0 female). EOA and LOA sample characteristics are detailed in Supplementary Table 1.

**TABLE 1:** Sample characteristics.

		Groups according to diagnostic outcome				*p*-value of Bonferroni’s multiple comparison		
	High-risk toddlers (*n* = 20)	HR-TD (*n* = 8)	HR-nonTD (*n* = 3)	HR-ASD (*n* = 9)	*p*-value	HR-TD / HR-ASD	HR-TD/HR-non-TD	HR-non-TD/HR-ASD
Mean MRI age [months] (*n* = 20)	16.5 ± 1.6 (14.6-19.8)	**17.7 ± 1.5 (15.6-19.8)**	**15.3 ± 0.5 (14.8-15.7)**	**15.8 ± 1.2 (14.6-18.3)**	**0.001**	**0.020**	**0.041**	
Time interval between scans [months] (*n* = 20)	6.5 ± 0.9 (5.2-9.0)	6.8 ± 1.4 (5.2-9.0)	6.1 ± 0.5 (5.6-6.6)	6.3 ± 0.5 (5.6-7.2)	0.474			
Gender (female number) (*n* = 20)	5	3	1	1	0.427			
Age at 1nd clinical assesment (*n* = 19)	18.1 ± 0.4 (17.6-19.1)	18.1 ± 0.4 (17.7-18.6)	17.8 ± 0.2 (17.6-18)	18.2 ± 0.4 (17.7-19.1)	0.253			
ADOS CSS (*n* = 19)	3.5 ± 2.7 (1-10)	2.6 ± 2.1 (1-7)	2 ± 1 (1-3)	4.8 ± 3.1 (1-10)	0.161			
DQ (*n* = 19)	87.1 ± 14.2 (54.1-119.4)	96.4 ± 14.9 (87.3-119.4)	85.8 ± 11.8 (72.6-95.5)	80.4 ± 11.2 (54.1-90.9)	0.073			
Age at 3rd clinical assesment (*n* = 20)	36.5 ± 1.2 (34.9-39.9)	36.9 ± 1.4 (35.6-39.9)	35.7 ± 0.7 (35.3-36.6)	36.9 ± 1.4 (34.9-38.3)	0.356			
ADOS CSS (*n* = 20)	3.9 ± 2.6 (1-8)	**1.5 ± 0.5** (1-2)	**2.7 ± 1.5** (1-4)	**6.3 ± 1.3** (5-9)	**<0.001**	**<0.001**		**<0.001**
DQ (*n* = 20)	87.5 ± 19.1 (70.8-108.0)	**103.2 ± 5.3 (93.3-109.9)**	**76 ± 0.2 (75.9-76.3)**	**77.4 ± 20.8 (32.5-103.2)**	**0.004**	**0.006**	**0.042**	

*Sample demographics and behavioral information. P-value of statistical comparisons between groups (One-way ANOVA) displayed as well as post hoc comparison between groups (Student’s t-test) with Bonferroni correction for multiple comparison when P-value was < 0.05. Significant differences between groups are highlighted in bold.*

One could notice that the proportion of ASD in our HR sample (45%) is greater than the prevalence of ∼20% which is reported in the literature (Ozonoff et al., 2011). Nevertheless, one must take into account the fact that our population is constituted by a majority of male HR in which the prevalence of ASD has been reported to be around 32% (Zwaigenbaum et al., 2012). Another explanation could rely in the fact that parents who were more worried about their child’s development were more motivated to participate to the scan acquisition, thus leading to a recruitment bias.

It should be noted that HR-TD do not share similar developmental trajectories with low-risk children with a typical development (LR-TD) as HR-TD exhibit higher ASD traits and increased risk for other conditions such as anxiety and attention-deficit/hyperactivity disorders (Charman et al., 2017; Shephard et al., 2017). As a supplementary analysis, we included the 10 LR-TD (2 females) from the cohort who completed two scans at 12–15 and 18–24 months for comparison with HR-TD and HR-ASD. The LR-TD mean age between two scans was 17.4 ± 1.9 months (15.4–20.1). Time interval between both scans was 7.1 ± 1.3 months (5.7–9.5).

### Behavioral Measures and Outcome Classification

Clinical assessments were conducted with each participant at 6, 12, 18, 24, and 36 months.

The Mullen Scales of Early Learning (MSEL) was used to assess development in cognitive (expressive and receptive language, visual reception) and motor (fine and gross) areas (Mullen, 1995). Developmental quotient scores (DQ) were used instead of standard scores in order to limit truncation of very low performing participants (Lord et al., 2006). Individual DQs were obtained by dividing age-equivalent developmental age output from MSEL by chronological age and multiplying by 100.

ASD-related symptom severity was quantified with the Autism Diagnostic Observation Schedule (ADOS) (Lord et al., 2002). The ADOS is a semi-structured observational evaluation with cut-offs to guide diagnostic decisions, appropriate for ambulatory children of 12 months and older. Either module 1 (intended for non-verbal children or those using only isolated words) or module 2 (intended for children with phrase speech) was conducted at ages 18, 24, and 36 months. To allow comparison of ADOS total scores across ages and modules, the calibrated severity score (CSS) was used. ADOS CSS ranges from 1 to 10 (with 10 being the most severe) (Gotham et al., 2009; Rutter et al., 2012).

At each visit from 18 months and later, ASD diagnostic outcome was established by a licensed clinician according to ADOS diagnosis cut-offs and DSM-IV criteria (American Psychiatric Association (APA), 2000). Children who did not meet the criteria for a diagnosis of ASD were categorized as having typical development (TD) or non-typical development (non-TD). TD was defined as having an ADOS CSS equal to or less than 2, a total DQ of at least 85, no DQ subtest less than 80 and no more than one DQ subtest less than 85. If one or more of these criteria were not met, participants without ASD were classified as non-TD.

### Image Acquisitions

All children were scanned during natural sleep following previously published procedures (Nordahl et al., 2008), at the UC Davis Imaging Research Centre on a 3 Tesla Siemens TIM Trio MRI system with an eight-channel head coil. Structural T1-weighted 3D MP-RAGE images were acquired with 1 mm^3^ isometric voxels, repetition time = 3,200 ms, echo time = 5.08 ms, field of view = 176 mm, and 192 sagittal slices. The success rate of these MRI acquisitions was 78%. A 3D image distortion map (Image Owl) was acquired at the end of each scan with a calibration phantom (Phantom Laboratory, Inc.). Distortion correction was carried out as described in Nordahl (2012).

Participants had a first MRI scan at 6–9 months of age which was not evaluated in the present analyses because of the difficulty to obtain accurate 3D white matter surface reconstructions at this age. Accordingly, we utilized the participant’s second scan, acquired between 12 and 15 months of age, and third scan, acquired between 18 and 24 months of age. This third scan was acquired an average of 1.5 ± 2.0 months after the 18-months ADOS.

### Image Processing and Quality Control

We used the automated pipeline provided by FreeSurfer v6.0 to process the T1-weighted cerebral MRIs^1^. The successive steps of this automated procedure are described in detail elsewhere (Dale et al., 1999; Fischl et al., 1999a, b; Fischl and Dale, 2000). Briefly, non-cerebral tissues are removed, signal intensity is normalized, and the image is segmented using a connected components algorithm. Then, a single filled volume of white matter is generated for each hemisphere. For each volume of white matter, a triangular surface tessellation is created by fitting a deformable template. Through deformation of this tessellated surface, a cortical mesh is created that defines the boundary between white and cortical gray matter (called the outer white matter surface) as well as the boundary between the gray matter and the extra-axial fluid (called the pial surface). This surface deformation process is calculated through an energy minimization function that determines the sharpest shift in intensity between voxels to define the transition between tissue categories. This process is independent of absolute intensity values and can delineate boundaries at a subvoxel resolution. The described pipeline has previously been applied within toddlers (Travis et al., 2014) as well as children with ASD (MRC Aims Consortium et al., 2018). Importantly, FreeSurfer delineation of white matter and gray matter is solely based on intensity shift and does not rely on an age specific template.

A trained operator (M.G.), blind to any clinical outcome, visually inspected images obtained with the described automated pipeline. First, he attributed a subjective score ranging from one to ten for every image denoting the level of motion artifact. A motion rating (MR) was then estimated for every participant by averaging these scores between their two scans. Second, he implemented manual corrections when required following recommended procedures described in the FreeSurfer manual^2^. All final cortical surfaces were visually validated by a second trained independent operator (M.S.) who was also blind to all clinical outcomes.

### Gray-White Matter Intensity Contrast

**FIGURE 1:**
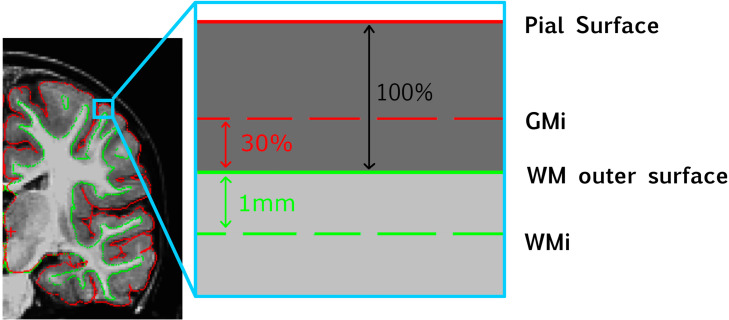
Schematic representation of how GWC is computed at each vertex. GMi, gray matter intensity; WM, white matter; WMi, WM intensity.

We first sampled white matter intensity at each vertex *v* at 1 mm beneath the white matter (WM) outer surface (Figure 1). A distance of 1 mm was chosen to facilitate comparisons with previous literature since it is the most commonly used value in GWC studies, including all existing studies exploring GWC in ASD (MRC Aims Consortium et al., 2018; Fouquet et al., 2019). Gray matter (GM) intensity value was sampled at each vertex at a distance of 30% of cortex width (defined as the distance between outer white matter surface and pial surface) starting from the white matter outer surface. The value of 30% was set because it is the most commonly used in the GWC literature (MRC Aims Consortium et al., 2018) and is the default value provided by FreeSurfer. In addition, a previous study of ASD found diagnostic differences to be greatest when GM intensity sampled between 30 and 40% was used to compute GWC (Fouquet et al., 2019). GWC at each vertex *v* was computed by dividing the difference between GM and WM intensities by the mean between GM and WM intensities and multiplying by 100 to get a ratio expressed in [%]. This was performed for each individual scan at time T.

(1) $${\text{GWC}}_{v}T\left[\%\right]=100\times \frac{\left(WM_{v}-GM_{v}\right)}{\left(WM_{v}+GM_{v}\right)/2}$$

GWC values were then registered on an average template provided by FreeSurfer to allow vertex-wise inter-participants comparison. During this process, GWC values were smoothed with a full-width at half-maximum (FWHM) surface-based Gaussian kernel of 10 mm.

Then, for each participant two different GWC longitudinal values were computed. Longitudinal neuroimaging designs allow many advantages over cross-sectional designs, including reduction of within-participant variability and the possibility to analyze the effect of time on the variable of interest (Reuter et al., 2012). First, we estimated the individual average GWC*v* values between 12 and 24 months of age by computing the mean of GWC*v* values between the two scans.

(2) $${\text{GWC}}_{v}\left[\%\right]=\frac{GWC_{v}1+GWC_{v}2}{2}$$

Where GWC*v*1 is GWC*v* at 12–15 months and GWC*v*2 is GWC*v* at 18–24 month.

Second, we computed the individual GWC rate of change between two scans (ΔGWC) at each vertex. ΔGWC represents the effect of time on GWC between the age of 12 and 24 months and was computed through the symmetrical percentage of change (SPC) formula (Reuter et al., 2012). SPC consists of calculating, for each participant at each vertex *v*, the GWC difference between two scans divided by the age difference between the two scans, giving a rate in [%/month]. This rate is divided by mean GWC at each vertex v and multiplied by 100, giving a result expressed in [%]. Using symmetrized measures of change (such as (B_y_−B_x_)/B_x_) over absolute differences (such as B_y_−B_x_ that would not be scaled to the mean) is recommended in longitudinal analyses as they allow many advantages such as increased statistical robustness, higher reliability in small samples and balanced consideration of both measures B_x_ and B_y_ (Berry and Ayers, 2006). SPC expresses the rate at which GWC changes in each vertex between two scans relative to mean GWC.

(3) $${\text{GWC}}_{v}\left[\%\right]=100\times \frac{\left(GWC_{v}2-GWC_{v}1\right)/\left(age2-age1\right)}{GWC_{v}}$$

Where *age1* is participant’s age at 12–15 months scan and *age2* is their age at 18–24 months scan.

### Cortical Thickness

Cortical thickness (CT) alterations have been found to influence GWC values (Westlye et al., 2009). CT is defined by the distance in mm between WM outer surface and pial surface and is automatically computed by the standard FreeSurfer processing pipeline (Fischl and Dale, 2000). To control for this possible confound, we sampled CT at each vertex *v*. We then computed the individual average CT*v* across the two scans and the CT*v* rate of change (ΔCT*v*) with the same formulas we described for longitudinal GWC parameters. Spatial overlap between significant effects on GWC and CT were explored.

### Motion Rating

Motion artifacts are a significant confound that can influence GWC (Fouquet et al., 2019). While we found no difference in motion rating (MR) across main outcome groups (HR-TD, HR-ASD and HR-non-TD), we also performed vertex-wise analyses to explore potential local correlations between MR and GWC. We found one cluster in the right parietal superior region with positive correlation between MR and GWC (CWP = 0.026, cluster size of 848.6 mm^2^), no significant results of interest were observed within this identified region.

### Statistical Analysis

#### Sample Characteristics Analyses

Our primary outcomes are symptom severity (ADOS CSS) at 18 and 36 months of age and discrete diagnosis at age 3 (HR-TD, HR-ASD, HR-non-TD). Mean age at scan acquisitions was 16.5 months (see Table 1). Accordingly we considered the evaluations performed at 18 months as the clinical correlate that was the closest in time to the neuroimaging data and thus refer to this as the phenotype at the time of scan acquisitions. Data derived from assessments at 36 months of age were used to test for associations between early neuroimaging parameters and later clinical outcomes.

We further subdivided the HR-ASD into late and early symptom onset (LOA and EOA). Associations between clinical outcome (HR-TD, HR-ASD and HR-non_TD) and individual characteristics that could represent potential confounding factors in GWC analysis were explored. These parameters included gender, age at scanning (which was calculated as mean age between two scans) and time interval between scans. According to the nature of the tested variables (i.e., discrete or continuous), we used either Pearson correlation, one-way ANOVA, Student’s *t*-test (or Mann-Whitney *U*-test when non-parametric distribution of variables was found), or chi-square test.

For descriptive purposes, we tested for differences in behavioral scores (DQ, ADOS CSS at 18 and 24 months) between diagnosis groups (HR-TD, HR-ASD, and HR-non-TD) using one-way ANOVA. We also tested for potential differences in behavioral scores between the two HR-ASD subgroups (EOA and LOA) using either Student’s *t*-test or Mann-Whitney *U*-test. Statistics described in this section were performed with Prism v.8.3.0 software with significance threshold set at alpha = 0.05. Behavioral and demographic characteristics of the sample are displayed in Table 1.

#### Surface-Based Analyses

We used the general linear model (GLM) command implemented in FreeSurfer to perform vertex-wise whole-brain surface-based analysis of GWC.

First, to determine the effect of time on GWC in typical development between the age of 12 and 24 months, we performed vertex-wise parametric comparison of ΔGWC values vs. zero in our HR-TD group. We then extracted vertex-wise ΔGWC values from all significant clusters and computed an average ΔGWC value for each hemisphere. This mean hemispheric ΔGWC value was compared between right and left to test for any asymmetry in the effect of age on GWC.

Then, we fit a GLM to test whether GWC and ΔGWC at age 12–24 months are associated with discrete diagnostic outcome at age 3 (HR-ASD or HR-TD). HR-non-TD were not included in this analysis in order to limit the number of group comparisons. Also, HR-non-TD does not represent a clearly distinct group with pure developmental delay but also includes children with autistic traits without a confirmed diagnosis. As such, HR-non-TD can be considered as an intermediate group in the symptom continuum between TD and ASD:

(4) $$\begin{array}{c} \text{GWC}∼Diagnosticoutcome+age1+age2\\ \Delta \text{GWC}∼Diagnosticoutcome+age1+age2 \end{array}$$

We further tested if symptom severity at 18 and at 36 months of age were associated with GWC and ΔGWC. All participants were included in this analysis. The following GLM was conducted:

(5) $$\begin{array}{c} \text{GWC}∼ADOSCSS+age1+age2\\ \Delta \text{GWC}∼ADOSCSS+age1+age2 \end{array}$$

Given that GWC before the age of 24 months is influenced by age (Travis et al., 2014), age at scanning was regressed out in all GLM analyses. The *p*-value for each voxel was calculated using two-tailed testing with significance threshold set at alpha = 0.05. Cluster-wise analyses were corrected for multiple comparisons using Monte-Carlo simulation (MCS) with a significance threshold for cluster-wise *p*-value (CWP) of alpha = 0.05. We used cluster-wise and MCS analysis pipelines implemented in FreeSurfer (Hagler et al., 2006).

The same GLM methods were utilized for analyses of CT and ΔCT values.

We then wanted to determine if potential alterations of GWC and ΔGWC found with GLM analysis were associated with the age at which the first ASD-related symptoms emerged. As a further *post hoc* exploratory analysis, for each cluster exhibiting significant GWC or ΔGWC alterations, we computed the average of all vertex-wise GWC or ΔGWC values, respectively, across all vertices in the cluster for each participant. These individual cluster-averaged GWC values were then compared between HR-TD, EOA and LOA using an ANCOVA with age at first scan and age at second scan as regressors. Significance threshold was set at alpha = 0.05. Whenever an ANCOVA reached significance, *post hoc* comparisons with multiple *t*-tests applying Bonferroni correction was performed.

## Results

### Sample Characteristics

Clinical characteristics of the sample are described in Table 1 and illustrated in Supplementary Figure 1. As expected, the HR-ASD group exhibited the most severe symptom severity and the lowest DQ at age 3. There was no significant difference between groups in either DQ or symptom severity at 18 months of age. This may be due to the fact that HR-ASD participants who already received an ASD diagnosis at this age (EOA participants) were too few (*n* = 4) to drive a statistically significant difference. A significant association between age at scanning and discrete diagnostic outcome groups was observed with HR-TD being elder than HR-non-TD and HR-ASD.

### HR With Typical Development Show Increased GWC Rate of Change Between Age 12 and 24 Months

**FIGURE 2:**
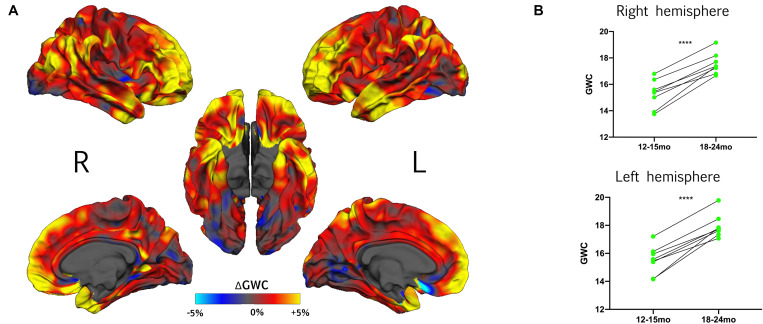
**(A)** Effect of time on GWC within HR toddlers with typical development outcome at 3 years (HR-TD) represented with vertex-wise ΔGWC values mapped on the common FreeSurfer template. **(B)** For each hemisphere, individual trajectories of gray-white matter contrast (GWC) from 12–15 to 18–24 months within the HR-TD group. Individual GWC displayed are the average of all vertex-wise values extracted from clusters with a significant effect of time on GWC (one per hemisphere, see Supplementary Figure 2). Right hemisphere: 12–15-mo GWC = 15.3% ± 1.1; 18–24 GWC = 17.6% ± 0.8. Left hemisphere: 12–15- months GWC = 15.5% ± 1.0; 18–24- months GWC = 17.9% ± 0.8. ****p < 0.0001 (Student’s *t*-test).

In HR children who demonstrated typical development at age 3 (HR-TD), GWC was significantly correlated with time in almost all regions (Figure 2 and Supplementary Figure 2). The highest rates of change were found bilaterally in prefrontal areas, temporal poles and temporo-parietal junctions. Areas with the smallest rates of change were found in lateral and medial occipital lobes bilaterally, right paracentral gyrus, right insula, left subgenual region and left inferior frontal gyrus. No regions exhibited a significant decreasing GWC rate of change. There was no global difference between right and left hemisphere (Supplementary Figure 2B).

### Increased GWC Between 12 and 24 Months of Age Is Associated With ASD at 36 Months

**FIGURE 3:**
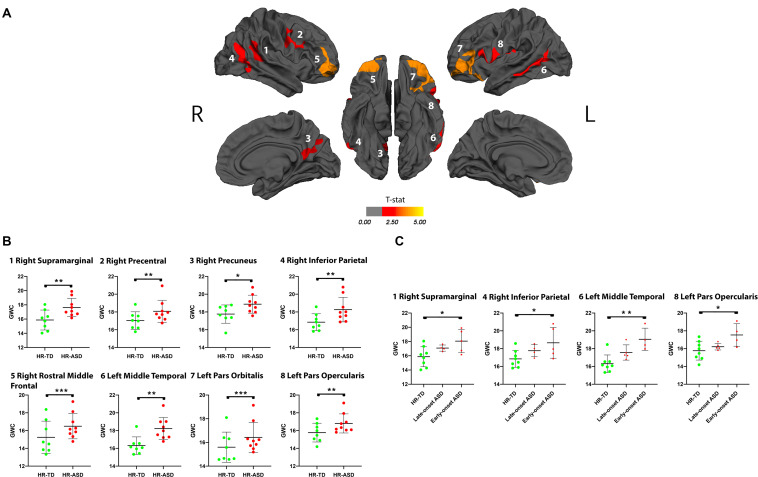
**(A)** Clusters in which gray-white matter contrast (GWC) at age 12–24 months (computed as mean GWC between the two scan acquisitions) has a significant association with diagnostic outcome at age 3 (HR-TD or HR-ASD). Only clusters with CWP < 0.05 after regression for age are and correction for multiple comparisons are displayed. Within each hemisphere, clusters are numbered in order of decreasing effect size (Cohen’s D, see Table 2). Color code corresponds to *p*-value of the vertex with the greatest *p*-value within the cluster. *P*-values are represented as T-stat = -log(*p*-value). **(B)** Individual mean GWC values for each significant cluster are displayed. **(C)** Comparison between HR-TD and HR-ASD subgroups (LOA and EOA). Only clusters with a significant ANCOVA (*p* < 0.05) are displayed. *p < 0.05, **p < 0.01, and ***p < 0.001.

A significant increase in GWC values in HR-ASD compared to HR-TD was found in the following regions: right supramarginal, right precentral, right precuneus, right inferior parietal, right rostral middle frontal, left middle temporal, left pars orbitalis and left pars opercularis (Figure 3). No regions displayed a significant decrease in GWC in HR-ASD compared to HR-TD (Table 2). There were no differences in terms of local ΔGWC between HR-ASD and HR-TD.

**TABLE 2:** Clusters of significant association between GWC and clinical outcomes.

mGWC	Cluster number	Anatomical label	Size (mm^2^)	CWP	Pvm	Effect size
Diagnostic outcome (HR-TD vs. HR-ASD)	1	Right supramarginal	886.4	0.009	3.432	0.748
	2	Right precentral	976.7	0.004	3.429	0.730
	3	Right precuneus	850.4	0.012	4.396	0.705
	4	Right inferior parietal	994.9	0.004	3.684	0.638
	5	Right rostral middle frontal	2013.0	<0.001	3.089	0.559
	6	Left middle temporal	1050.3	0.003	3.682	0.757
	7	Left pars orbitalis	2733.2	<0.001	4.133	0.659
	8	Left pars opercularis	985.8	0.002	4.646	0.651
18 months ADOS CSS	1	Right inferior parietal	713.7	0.038	4.127	0.835
	2	Left middle temporal	803.6	0.015	3.510	0.758
	3	Left pars opercularis	982.1	0.003	4.016	0.603
36 months ADOS CSS	1	Right precentral	785.6	0.022	2.595	0.787
	2	Right precuneus	899.8	0.007	4.648	0.775
	3	Right inferior parietal	728.0	0.034	3.669	0.722
	4	Right lateral occipical	1342.2	<0.001	3.283	0.562
	5	Left paracentral	727.2	0.028	3.215	0.861
	6	Left lateral occipital	1346.8	<0.001	3.303	0.712
**ΔGWC**						
18 months ADOS CSS	1	Right pars opercularis	1662.9	<0.001	3.334	0.835
	2	Right superior frontal	806.9	0.018	2.865	0.766
	3	Right inferior parietal	1232.7	<0.001	2.506	0.759
	4	Left posterior cingulate	1240.9	<0.001	3.631	0.899
	5	Left superior parietal	840.9	0.011	5.019	0.840
	6	Left supramarginal	1536.1	<0.001	2.915	0.814
	7	Left superior frontal	1280.9	<0.001	3.640	0.790
	8	Left middle temporal	905.0	0.007	3.097	0.746

**FIGURE 4:**
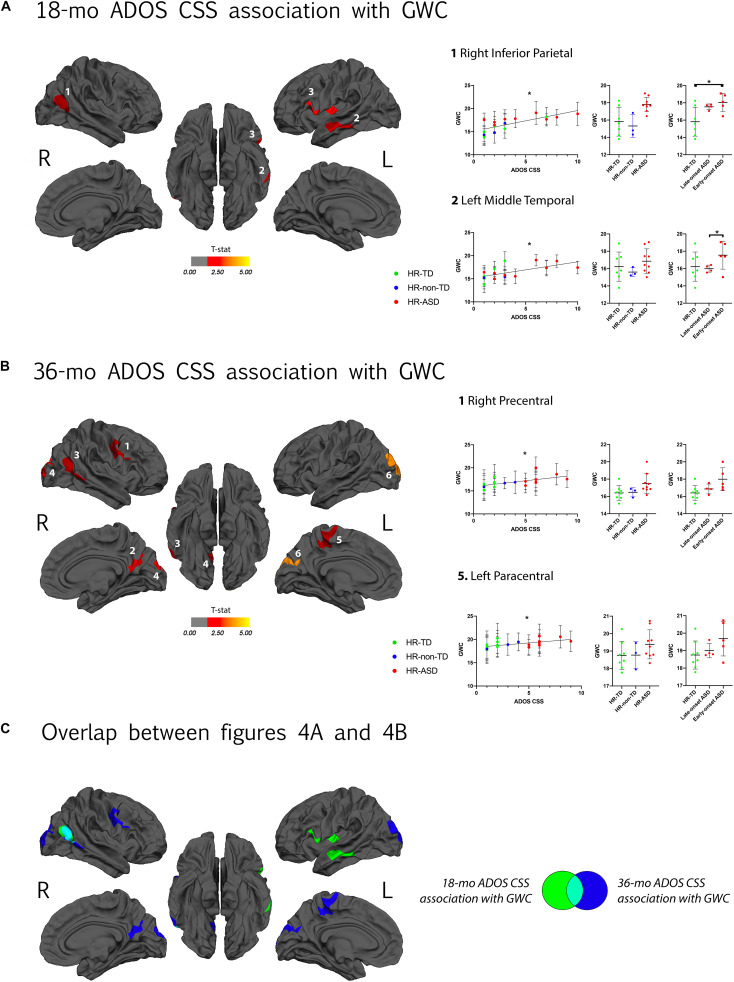
**(A)** Clusters with a significant association between gray-white matter contrast (GWC) at age 12–24 months (computed as mean GWC between two scan acquisitions) and ADOS calibrated severity score (CSS) at 18 months of age. Only clusters with CWP < 0.05 after regression for age and correction for multiple comparisons are displayed. Color code corresponds to *P*-value of the vertex with maximal *p*-value (Pvm) of each cluster and is represented as T-stat values (see Table 2). Within each hemisphere, clusters are listed in order of decreasing effect size (Pearson’s R, see Table 2). On the right, individual mean GWC values are displayed in function of ADOS CSS for clusters with the greatest effect size. Same individual values are further plotted in function of diagnostic outcome group (HR-TD, HR-non-TD, and HR-ASD) in the middle graph. In the right graph, comparison between HR-TD and HR-ASD subgroups (LOA and EOA) are displayed. Similar graphs for all significant clusters are available in Supplementary Figure 3A. **(B)** Association between GWC at age 12–24 months of age and symptom severity at 36 months of age. Detailed results for all clusters are displayed in Supplementary Figure 3B. **(C)** Clusters of **(A,B)** displayed on a common template with color code corresponding to the age of clinical assessment (green for 18 and blue for 36 months of age). *p < 0.05.

**FIGURE 5:**
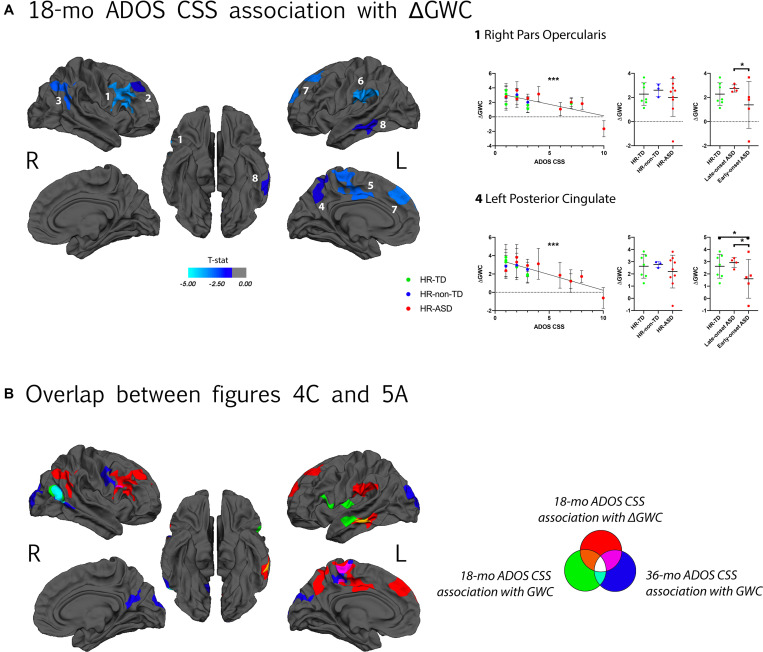
**(A)** Association between GWC rate of change (ΔGWC) between 12 and 24 months of age and ADOS calibrated severity score (CSS) at 18 months of age. Only clusters with CWP < 0.05 after regression for age and correction for multiple comparisons are displayed. Color code corresponds to *P*-value of the vertex with maximal *P*-value (Pvm) of each cluster (see Table 2). In each hemisphere, clusters are listed in order of decreasing effect size (Pearson’s R, see Table 2). Individual ΔGWC are displayed in function of ADOS CSS on the graph on the left. Only values for clusters with the greatest effect sizes are shown. Same individual values are further plotted in function of diagnostic outcome group (HR-TD, HR-non-TD and HR-ASD) in the middle graph. On the right, comparison between HR-TD and HR-ASD subgroups (LOA and EOA) are displayed. Similar graphs for all significant clusters are displayed in Supplementary Figure 4. **(B)** Clusters displayed on **(A)** and clusters with a significant association between GWC at age 12–24 months and ADOS CSS at 18 months and 36 months of age (Figure 4C) displayed on a common template. Color code reflects which longitudinal GWC value (either mean GWC or ΔGWC) and which ADOS CSS (either at 18 months or 36 months of age) were used in GLM. *p < 0.05, **p < 0.01, and ***p < 0.001.

*Detailed results of surface-based statistical analyses. Cluster numbers refer to numbers displayed in Figures 3–5 and are listed in order of decreasing effect size within each analysis. Anatomical labels refer to location of vertex with maximal p-value (Pvm) according to FreeSurfer Desikan parcellation atlas. Pvm are reported in T-stat values which is equal to -log(p-value). Effect sizes are expressed with either Cohen’s d or R squared (r^2^) values. CWP, Cluster-wise p-value.*

### Increased GWC Between 12 and 24 Months of Age Is Associated With ASD Symptom Severity at 18 and 36 Months

GWC was positively correlated with autism symptom severity (ADOS CSS) at 18-months in the following cortices: right inferior parietal, left middle temporal and left pars opercularis (Figure 4A). GWC was also positively correlated with autism symptom severity at age 3 (36-months ADOS CSS) in the right precentral, right precuneus, right inferior parietal, right lateral occipital, left paracentral and left lateral occipital regions (Figure 4B and Table 2). All clusters are illustrated in Supplementary Figure 3.

### Slower GWC Rate of Change Between Age 12 and 24 Months Is Exclusively Associated With Symptom Severity at 18 Months

A negative correlation between symptom severity (ADOS CSS) at 18-mo and ΔGWC values was observed in the right pars opercularis, right superior frontal, right inferior parietal, left posterior cingulate, left superior parietal, left supramarginal, left superior frontal, and left middle temporal areas (Figure 5A). That is, higher symptom severity at 18 months was associated with slower ΔGWC between 12 and 24 months in these regions. Later symptom severity (36-mo ADOS CSS) as well as diagnosis group comparison (HR-TD vs. HR-ASD) were not associated with any significant differences in ΔGWC between age 1 and 2. See Table 2 and Supplementary Figure 4 for detailed results and illustrations.

### Alterations in GWC Values Are Influenced by the Age of First Reliable ASD Diagnosis

We performed a further exploratory *post hoc* analyses on HR-ASD subgroups based on whether ASD diagnosis was established at 18 months (EOA) or later (LOA). We found that the clusters with altered GWC values in HR-ASD were mostly driven by participants with an early diagnosis onset. In clusters with higher GWC values in HR-ASD compared to HR-TD the EOA subgroup exhibited increased GWC values compared to HR-TD in right supramarginal, right inferior parietal, left middle temporal and left pars opercularis. The LOA subgroup didn’t exhibit any clusters of significantly increased GWC values compared to HR-TD (Figure 3).

Among brain regions indicated as having significant correlations between GWC and symptom severity at 18-months, there were significant differences in GWC between EOA and HR-TD in the right inferior parietal cortex and between EOA and LOA (EOA having greater values) in the left middle temporal gyrus. There were no differences in GWC between LOA and HR-TD (Figure 4A and Supplementary Figure 3). For clusters with significant correlations between GWC and 36 months symptom severity (36-mo ADOS CSS), we found significant differences in GWC between EOA and HR-TD in the right inferior parietal region (Figure 4B and Supplementary Figure 3).

For clusters with significant correlation between symptom severity at time of scan and ΔGWC significant differences were found in ΔGWC between EOA and LOA in all clusters except those incorporating right inferior parietal and left supramarginal regions. Moreover, EOA exhibited slower ΔGWC compared to HR-TD in the left posterior cingulate and left superior parietal regions. There were no differences between HR-TD and LOA in any of the clusters (Figure 5 and Supplementary Figure 4).

### Cortical Thickness at 12–24 Months Is Decreased in Medial Superior Frontal Gyrus in the HR-ASD Group

We found decreased CT values in HR-ASD compared to HR-TD in a single cluster located in the left superior frontal region (cluster size of 679.7 mm^2^, cluster-wise *p*-value of 0.042, see Supplementary Figure 5). No differences in ΔCT between HR-ASD and HR-TD were observed. There was also no effect of symptom severity (either 18-months or 36-months ADOS CSS) on either CT or ΔCT.

### LR-TD Comparison With HR-TD and HR-ASD

As a supplemental analysis, we performed vertex-wise comparisons of GWC and ΔGWC between LR-TD and HR-TD, as well as between LR-TD and HR-ASD. We found that HR-ASD have higher GWC in the left supramarginal area compared to LR-TD (CWP = 0.018, cluster size of 778.6 mm^2^). We also found that HR-TD have lower GWC in the right lateral orbitofrontal cortex compared to LR-TD (CWP = 0.045, cluster size of 685.2 mm^2^). There was no difference in terms of ΔGWC. Results are displayed in Supplementary Figure 6. ΔGWC values within the LR-TD are displayed in Supplementary Figure 7.

## Discussion

Our aim was to use structural MRI to conduct an exploratory surface-based analysis to determine if alterations in tissue contrast across the gray-white matter boundary in toddlers at high-risk for ASD could represent an early biomarker of clinical outcome (i.e., autism diagnosis) at age 3 and whether alterations in GWC are associated with autism symptom severity. To our knowledge, this is the first study to explore GWC in children aged less than 24 months old who are at a high risk to develop ASD. Firstly, HR children with typical development were found to exhibit widespread increases of GWC values with time from ages 12 to 24 months. This result provides a first normative reference for typical GWC values at this age in HR toddlers. Secondly, ASD outcome at 3 years of age was associated with widespread (though well localized) increased GWC values during the second year of life compared to HR infants with a TD outcome. These results suggest that brain microstructural alterations in ASD are already present at the end of infancy and are associated with clinical outcomes later in development. Lastly, individuals who experienced more severe symptoms of ASD at 18 months of age showed a distinct neurobiological signature characterized by a slower rate of change in GWC between 12 and 24 months of age.

### Typical Development in a HR Population Is Characterized by Increasing GWC Values Between 12 and 24 Months of Age

The only previous study exploring GWC changes in TD across the same age range as the current study also identified brain regions where GWC values increased between age 12 and 19 months (Travis et al., 2014). Clusters found by Travis et al. (2014) correspond to regions observed in the current study with the highest ΔGWC values in the HR-TD group (left dorso-lateral prefrontal cortex and left anterior temporal lobe). However, Travis et al. (2014) found more focal regions with significant increases in ΔGWC in comparison to our results and exclusively localized in the left hemisphere. Several possible explanations may explain these differences. One is the exploration of a shorter age interval by Travis et al. (2014) (from 12 to 19 months instead of 12 to 24 months in our study) which may result in reduced effects of time on GWC. Another explanation is the more conservative method to correct for multiple corrections used by Travis et al. (2014) (false discovery rate instead of MCS). Nonetheless, collectively these results converge in supporting the idea that GWC tends to increase with time in various regions during the second year of life in TD.

### Relations Between Our Results and GWC Alterations Previously Found at Older Ages

Considering existing literature exploring GWC in ASD, GWC differences in regions reported as altered in older populations with ASD were also observed in the current study. For instance, bilateral middle temporal gyri (MTG) exhibited decreased GWC in Andrews et al. (2017). Furthermore, right precuneus, right occipital gyri and left inferior frontal gyrus (IFG) exhibited decreased GWC values in association with ASD from late childhood to early adulthood in MRC Aims Consortium et al. (2018). Although brain regions indicated by the current study are consistent with these two previous studies of adults, we found that ASD was associated with increased GWC. Although inconsistent, these observations are not necessarily incompatible. One potential explanation is the possibility that between the age explored in our study (i.e., before 2 years of age) and later ages, the GWC rates of change are slower amongst children with ASD compared to TD. This hypothesis is supported by our finding that the second year of life is characterized by slower GWC rates of change in individuals who experienced more severe symptoms of ASD during the period of scan acquisitions. Further studies exploring GWC trajectories in later childhood are needed to confirm that ASD is characterized by slower GWC rates of change at some point as we did not find any group difference in ΔGWC between HR-ASD and HR-TD in our sample.

Some of our results were consistent in location and direction with previous literature, e.g., increased GWC in bilateral occipital and left paracentral gyri was previously described by Fouquet et al. in adults with ASD (Fouquet et al., 2019). This suggests that increased GWC in primary cortices (visual cortex and primary motor areas) could remain higher across lifespan in individuals with ASD compared to TD. Also, regions found to have slower GWC changes with time (ΔGWC) were consistent with areas described to have later decreased GWC in Mann et al. (for bilateral prefrontal and parietal posterior cortices) and Andrews et al. (2017) (for left MTG, left precuneus, and left medial part of superior frontal gyrus).

### Increased GWC at 12–24 Months of Age Relates to ASD Outcome at 36 Months of Age

Areas in which increased GWC were associated with ASD diagnosis at age 3 (Figure 3) have all been previously implicated in functions altered in ASD, including language and social processing (Amaral et al., 2008). Left middle temporal gyrus (MTG) and left inferior frontal gyrus (IFG), for instance, are both implicated in semantic processing which is one of the most commonly found altered domains of language in ASD (Tager-Flusberg et al., 2006; Huang et al., 2012; Wei et al., 2012). Moreover, left IFG is known to be functionally altered during semantic processing tasks in adults with ASD (Harris et al., 2006). Left MTG and left IFG both exhibit morphometric alterations in adults with ASD (Libero et al., 2014). Right MTG has shown metabolic activation related to multimodal integration of communication cues (i.e., gaze, speech and gesture) (Holler et al., 2015) in TD, processes known to be challenging for individuals with ASD (Stevenson et al., 2014). The right precuneus is a key component of the default mode network (DMN), which is implicated in mentalizing (i.e., building inferences about others’ mental states). Such “theory of mind” deficits have been highlighted as a feature of ASD for decades (Baron-Cohen et al., 1985) and there is growing evidence supporting the presence of alterations in the DMN and more specifically precuneus in ASD (Padmanabhan et al., 2017).

Regarding regions in which high GWC values at age 12–24 months were associated with symptom severity at age 3 (Figure 4B), some overlap with clusters associated with later diagnosis outcomes are present, such as right precuneus, and right MTG. Nonetheless, some clusters were exclusively associated with symptom severity at age 3 and not later diagnosis outcome. One possible explanation of this apparent discrepancy is the fact that HR-non-TD were excluded from GWC comparison between diagnosis outcomes. HR-non-TD comprises children with borderline ADOS scores reflecting children with some autistic traits but not expressing the full phenotype. It is thus possible that the adjunction of HR-non-TD in the ADOS correlation analyses provided enough statistical power to drive a significant correlation between ADOS and GWC in these clusters that was otherwise missed in the binary outcome comparison.

Another explanation is that some HR-TD present mild autistic traits (i.e., ADOS calibrated severity scores of 2). This heterogeneity within HR-TD is not taken into account in discrete group comparisons but is included within the ADOS correlation analyses. Increased GWC values in primary cortices such as the occipital gyri (visual) and precentral gyrus (motor) in association with symptom severity is consistent with the results reported by Fouquet et al. (2019) on GWC in adults with ASD. It is also consistent with previous reports of disruption of primary motor area organization in children with ASD (Nebel et al., 2014) as well as functional and structural alterations in occipital regions in the same population (Jung et al., 2019). Our results suggest that a common microstructural mechanism during the first years of life could be at play across various cortical areas that all have been independently reported as functionally and/or structurally altered in ASD. Finally, regions exhibiting increased GWC values in relation to symptom severity at 18-months (Figure 4A) are mostly overlapping with regions that are associated with later ASD diagnoses (IFG, left MTG) or later symptom severity (right MTG).

### Slower GWC Rate of Change Between 12 and 24 Months of Age as a Neural Signature of the ASD Symptom Severity at the Age of 18 Months

A widespread decrease in the rate of GWC change between 12 and 24 months of age was associated with ASD symptom severity at 18 months. Most clusters with slower ΔGWC did not overlap with regions that had increased GWC associated with diagnostic outcome at age 3 (Figure 5B). ΔGWC alterations were largely localized within the central executive network (CEN), including bilateral dorso-lateral prefrontal and bilateral posterior parietal cortex (Fox et al., 2006). Functional alterations of CEN have previously been reported in ASD (Perez Velazquez et al., 2009). Decreased GWC rate of change was also observed in left temporo-parietal junction (TPJ), left precuneus and left middle temporal gyrus (MTG). The TPJ and precuneus are both within the DMN which has been found altered in ASD (Padmanabhan et al., 2017). Furthermore, the left MTG exhibits both increased GWC and decreased ΔGWC between age 12 and 24 months. This alteration of left MTG microstructure further supports its possible role as a key region in the early development of ASD phenotypes. Overall, these results suggest that participants who manifest early symptoms of ASD are characterized by a specific pattern of dynamic GWC changes during the second year of life, largely affecting regions implicated in executive functions.

### Neurobiological Interpretations of Altered GWC Values

To understand the neurobiological correlates to our findings, an important step would be to explore if GWC differences result from alterations in cortical gray matter (GM), superficial white matter (WM), or a combination of both structures. Since GWC measures depend on two parameters (WM intensity and GM intensity), changes in GWC values can be caused either by changes in one or both of these variables. The GWC measure is proportional to WM intensity and inversely proportional to GM intensity. In other words, a darker gray matter intensity and a brighter superficial white matter intensity would both result in increased GWC values. The opposite reasoning holds for explaining decreased GWC values. Unfortunately, in qualitative MRI techniques such as T1-weighted scans, the absolute intensity values can be influenced by many factors (type of setup, detectors used, etc.) resulting in great intra- and inter-participants variability (Lutti et al., 2014). It is thus of limited utility to perform statistical analyses on GM and/or WM intensities *per se*, an issue that was already highlighted in early studies using GWC (Westlye et al., 2009).

Despite this intrinsic limitation, we can speculate here on the likelihood of various neurobiological correlates which could explain enhanced GWC values during the second year of life. First, increased GWC could result from decreased GM intensity (i.e., a darker cortical gray matter on T1w images). Many studies have highlighted alterations of cortical cytoarchitecture in ASD which could lead to alterations in GM intensity. For instance, greater density of dendritic spines of pyramidal cells as well as an increased neural density have been reported in various cortical regions and in the amygdala amongst children and adults with ASD (Avino et al., 2018). Evidence suggests that an increased number of minicolumns (which constitute the basic structural units of cortical architecture) could be a potential cause of neural excess in ASD (Casanova et al., 2006). If our results are explained by increased neural density at the end of infancy they would thus bring further support to the hypothesis that ASD is characterized by altered neural proliferation, migration and lamination processes (Packer, 2016). Another explanation for decreased GM intensity would be a delay in intracortical myelination (Fouquet et al., 2019). Nevertheless, deficits in intracortical myelin are not well documented in ASD and limited to animal model studies (van Tilborg et al., 2017).

Increased WM intensity represents an alternative (although not exclusive) explanation to increased GWC. If this were the case, increased myelination would be a likely contributor, since myelin is the most determinant contribution to WM intensity (Koenig, 1991). Early increased myelination in ASD has been suggested by several studies exploring white matter tracts in infancy using diffusion weighted imaging (Wolff et al., 2012, 2015; Solso et al., 2016; The IBIS Network et al., 2017b; Andrews et al., 2019). If true, these results would support the idea that ASD is characterized by an increased myelination processes during the first months of life.

Potential mechanisms underlying decreased GWC rates of change include faster increases in GM intensity (i.e., cortical gray matter becoming rapidly brighter) and/or slower increases in WM intensity (i.e., superficial white matter becoming slowly brighter). Slower WM intensity changes could be explained by a delay in myelination of superficial WM. This hypothesis would be consistent with previous reports of decreased myelin in superficial WM in adolescent and adults with ASD (Hong et al., 2019). Furthermore, this hypothesis would converge with previous studies who showed an early developmental pattern that could be indicative of increased myelin content in various WM tracts during infancy followed by a delayed myelination process after the age of 12 months in ASD compared to TD (Wolff et al., 2012; Solso et al., 2016; Andrews et al., 2021). Thus, decreased myelin in superficial WM could be part of a more generalized alteration of the myelin as Ameis and Catani (2015) concluded that adults with ASD present a globally decreased connectivity after a review of diffusion imaging literature.

To overcome limitations in the biological interpretation of GWC measures, future studies would benefit from implementing MRI techniques that offer a quantitative measure of the local intensity to decipher respective contributions of cortical gray matter and superficial white matter to GWC alterations. One solution could be the use of imaging methods that precisely “map” the physical T1 or T2 properties of the tissue to allow local quantification and inter-participants comparison of microstructure content (Marques et al., 2010; Hilbert et al., 2018).

### GWC Alterations as a Specific Neurobiological Signature of ASD Diagnosis and Symptom Severity at 18 Months of Age

The current findings indicate that differences in GWC at 12–24 months are related to age at which first ASD symptoms occur. First, symptom severity at 18 months (i.e., ADOS CSS at 18 months) was associated with a pattern of GWC alterations that was distinct from alterations associated with later clinical outcome (ADOS CSS and diagnosis outcome at 36 months). E.g., slower GWC rates of change were specifically observed in relation to symptom severity at time of scan and were not linked to any later clinical outcome. Second, our *post hoc* exploratory analyses found that the subset of HR-ASD with established diagnosis at 18 months (i.e., early onset autism) exhibited the greatest magnitude in GWC alterations within all clusters across all analyses compared to HR-ASD with a later onset of ASD after 18 months (Supplementary Figures 3, 4). Since the early and late onset subgroups did not exhibit any difference in either symptom severity or global development (DQ) at age 3 (see Table 1), these differences can solely be explained by age of first reliable diagnosis onset and not by later symptom severity or cognitive level. Together, these results suggest that children with ASD experiencing more severe symptoms and a reliable diagnosis at 18 months are characterized by a specific pattern of early GWC alterations at the age of 1–2 years which consists of widespread slower GWC rate of change and a trend for all GWC alterations to be greater in magnitude in comparison to the rest of individuals with ASD.

### Minor Differences Between LR-TD and HR-ASD

Compared to our analysis performed within the HR population, differences between LR-TD and HR groups (HR-TD and HR-ASD) were scarce. HR-ASD exhibit one cluster with increased GWC compared to LR-TD in the left supramarginal region. This suggests that higher GWC is a specific signature of HR-ASD compared to both LR-TD and HR-TD. The reason why we found so few differences in GWC between HR-ASD and LR-TD are not clear. One could hypothesize that HR-ASD and HR-TD would be less different than HR-ASD and LR-TD since HR children share some common genetic risk together and have some similarities in their development (Messinger et al., 2013). Our results highlight that HR-TD have a different neurodevelopmental trajectory compared to HR-ASD in the early years. This could be caused by neural compensatory mechanisms that seek to counter neural predisposition to ASD. Our results also highlight the fact that HR-TD and LR-TD represent distinct population in terms of neurodevelopment and should be analyzed separately.

## Limitations and Further Perspectives

Some limitations to our study need to be highlighted. First is the small size of our sample that limits our study to an exploratory purpose. Indeed, small samples have been shown to be associated with a lower degree of replicability in functional MRI (Turner et al., 2018; Grady et al., 2021) as well as in structural MRI studies (Katuwal et al., 2015; Schaer et al., 2015). This limitation especially holds for our *post hoc* analyses on ASD subgroups. We nevertheless considered that these subgroup analyses offered an interesting deciphering of our main results and provide interesting hypotheses on which future research can build upon. Replication with larger samples will be necessary, especially to better delineate different phenotypic subgroups according to their distinct GWC alterations.

Second, motion artifacts (Fouquet et al., 2019) as well as alterations of cortical thickness could represent confounding factors to our results (Westlye et al., 2009). Nevertheless, vertex-wise analyses only revealed a single focal cluster with decreased mean cortical thickness in the HR-ASD group. The vast majority of regions found to have altered GWC or ΔGWC values in relation to ASD showed no significant differences in cortical thickness. Additionally, motion in our sample was only correlated with GWC in the right superior parietal region and did not overlap with any cluster from our main results. We can thus reasonably rule out alterations of cortical thickness as well as motion as confounding factors.

Finally, one could wonder how observed alterations are specific to ASD and not related to broader developmental delay. In our sample, HR-ASD exhibited a significant global delay (*DQ* = 77.4 in average, see Table 1) and it is impossible to exclude that this delay was in part responsible for the observed differences. Further studies including either a group of children with developmental delay without ASD or children with ASD without developmental delay are needed to address this limitation.

## Conclusion

In conclusion, our results support the hypothesis that ASD is associated with widespread microstructural alterations at the gray-white matter boundary during the first 2 years of life. These alterations were linked to symptom severity at 18 months of age, and also with later diagnosis outcomes and symptom severity at 3 years of age. GWC alterations in ASD consisted of increased contrast across many brain regions relevant for social processing, language acquisition as well as in primary visual and motor cortical regions. In parallel, children who experienced more severe symptoms of autism at 18 months of age exhibited slower GWC rates of change during the second year of life in many regions that are important for attentional and executive processing. Finally, all the GWC alterations that we reported were globally stronger in toddlers who already received a reliable ASD diagnosis at 18 months compared to those who developed ASD later. A potential neurobiological explanation of these findings might involve delayed myelination of superficial white matter, a hypothesis which will need to be assessed by further quantitative neuroimaging studies. Together, our results suggest that early enhancement of GWC in many regions is associated with later autism diagnosis and symptom severity, and that autism symptom severity at the age of 18 months is associated with a specific corresponding early developmental brain signature.

## Data Availability Statement

The raw data supporting the conclusions of this article will be made available by the authors, without undue reservation.

## Ethics Statement

The studies involving human participants were reviewed and approved by the University of California at Davis Institutional Review Board. Written informed consent to participate in this study was provided by the participants’ legal guardian/next of kin.

## Author Contributions

SO, DGA, and CWN conceived of and designed the study and acquired all clinical and neuroimaging data. DGA, JL, and GY provided technical assistance. MG prepared and analyzed the data under the supervision of MS and CWN. MG wrote the manuscript with the input from all other authors. All authors participated in interpretation of results, read and approved the final manuscript.

## Conflict of Interest

The authors declare that the research was conducted in the absence of any commercial or financial relationships that could be construed as a potential conflict of interest.

## Footnotes

1 http://surfer.nmr.mgh.harvard.edu/

2 http://freesurfer.net/fswiki/

## Supplementary Material

Supplementary Figure 1 Graphic representation of individual ADOS CSS at 18 and 24 months of age in our sample (*n* = 20 HR participants). Color code represents individual diagnosis outcome at age 3. HR-TD: high risk for ASD with typical development; HR-non-TD: high risk for ASD with atypical development; HR-ASD: high risk for ASD with ASD.

Supplementary Figure 2 **(A)** Clusters with a significant effect of time on gray-white matter contrast (GWC) in the HR-TD group. Clusters with cluster-wise *P*-value (CWP) < 0.05 only are displayed. Color code corresponds to *P*-value of the vertex with the maximal *P*-value (Pvm) of each cluster. **(B)** On the right are plotted for each hemisphere the individual GWC rates of change (ΔGWC) within each significant cluster (one per hemisphere). We found no significant difference between both clusters (paired *t*-test, *p* = 0.15).

Supplementary Figure 3 Results displayed on Figure 4 (Association between GWC at age 12–24 months and symptom severity at 18 months of age) with individual GWC values displayed for each significant cluster in function of ADOS calibrated severity score (CSS) (upper-side graphs), diagnostic outcome group (left down-side graphs) and HR-ASD subgroups (right down-side graphs). **(A)** Results for clusters with significant correlation between GWC and 18-mo ADOS CSS. **(B)** Clusters with significant correlation between GWC and 36-mo ADOS CSS. ^∗^*p* < 0.05, ^∗∗^*p* < 0.01, and ^∗∗∗^*p* < 0.001.

Supplementary Figure 4 Results displayed on Figure 5 (Association between symptom severity at 18 months of age and GWC rate of change (ΔGWC) between 12 and 24 months of age) with individual ΔGWC displayed for each cluster in function ADOS calibrated severity score (CSS) (upper-side graph), diagnostic outcome group (left down-side graphs) and HR-ASD subgroups (right down-side graphs). ^∗^*p* < 0.05, ^∗∗^*p* < 0.01, and ^∗∗∗^*p* < 0.001.

Supplementary Figure 5 Association between cortical thickness at age 12–24 months and diagnostic outcome at 36 months of age (HR-ASD or HR-TD). The single cluster with significantly smaller CT in HR-ASD compared to HR-TD (CWP < 0.05) is displayed. We found no significant cluster in the right hemisphere. Color code corresponds to *P*-value of the vertex with maximal *P*-value (Pvm) of the displayed cluster. On the right, individual *CT*-values are displayed in function of diagnosis outcome for the displayed cluster. ^∗^*p* < 0.05. CWP, cluster-wise *P*-value.

Supplementary Figure 6 Results of supplementary analysis comparing LR-TD with HR-ASD **(A)** and LR-TD with HR-TD **(B)**. Color code corresponds to *P*-value of the vertex with maximal *P*-value (Pvm) of the displayed clusters. On the graphs, individual GWC values are displayed in function of diagnosis outcome for the displayed clusters.

Supplementary Figure 7 **(A)** Effect of time on GWC within LR toddlers with typical development outcomes at 3 years (LR-TD) represented with vertex-wise ΔGWC values mapped on the common FreeSurfer template. **(B)** Clusters with a significant effect of time on gray-white matter contrast (GWC) in the LR-TD group. Clusters with cluster-wise *P*-value (CWP) < 0.05 only are displayed. Color code corresponds to *P*-value of the vertex with the maximal *P*-value (Pvm) of each cluster.

Supplementary Table 1 Demographic and behavioral information on HR-ASD subgroups (LOA and EOA). *P*-value of statistical comparison between both subgroups.

## Abbreviations

ADOS: Autism Diagnostic Observation Schedule
ASD: Autism Spectrum Disorder
CEN: central executive network
CSS: calibrated severity score
CT: Cortical thickness
CWP: Cluster-wise *p*-value
DMN: default mode network
DQ: Developmental quotient
EOA: Early onset ASD
FWHM: Full-width at half-maximum
GLM: General linear model
GM: Gray matter
GWC: Gray-white matter contrast
HR: High risk for ASD
HR-non-TD: HR with atypical development
HR-TD: HR with TD
IFG: Inferior frontal gyrus
LOA: Late onset ASD
LR-TD: Low risk for ASD with TD
MCS: Monte-Carlo simulation
MRI: Magnetic resonance imaging
MSEL: Mullen Scales of Early Learning
MTG: Middle temporal gyrus
Pvm: vertex with maximal *p*-value
SPC: Symmetrical percentage of change
TD: Typical development
TPJ: temporo-parietal junction
WM: White matter
Δ CT: CT rate of change
Δ GWC: GWC rate of change.
