# Supplementary figures and images for: Altered Gray-White Matter Boundary Contrast in Toddlers at Risk for Autism Relates to Later Diagnosis of Autism Spectrum Disorder
Source: Front Neurosci. 2021 Jun 17;15:669194. doi: 10.3389/fnins.2021.669194 (PMC8248433; doi:10.3389/fnins.2021.669194)

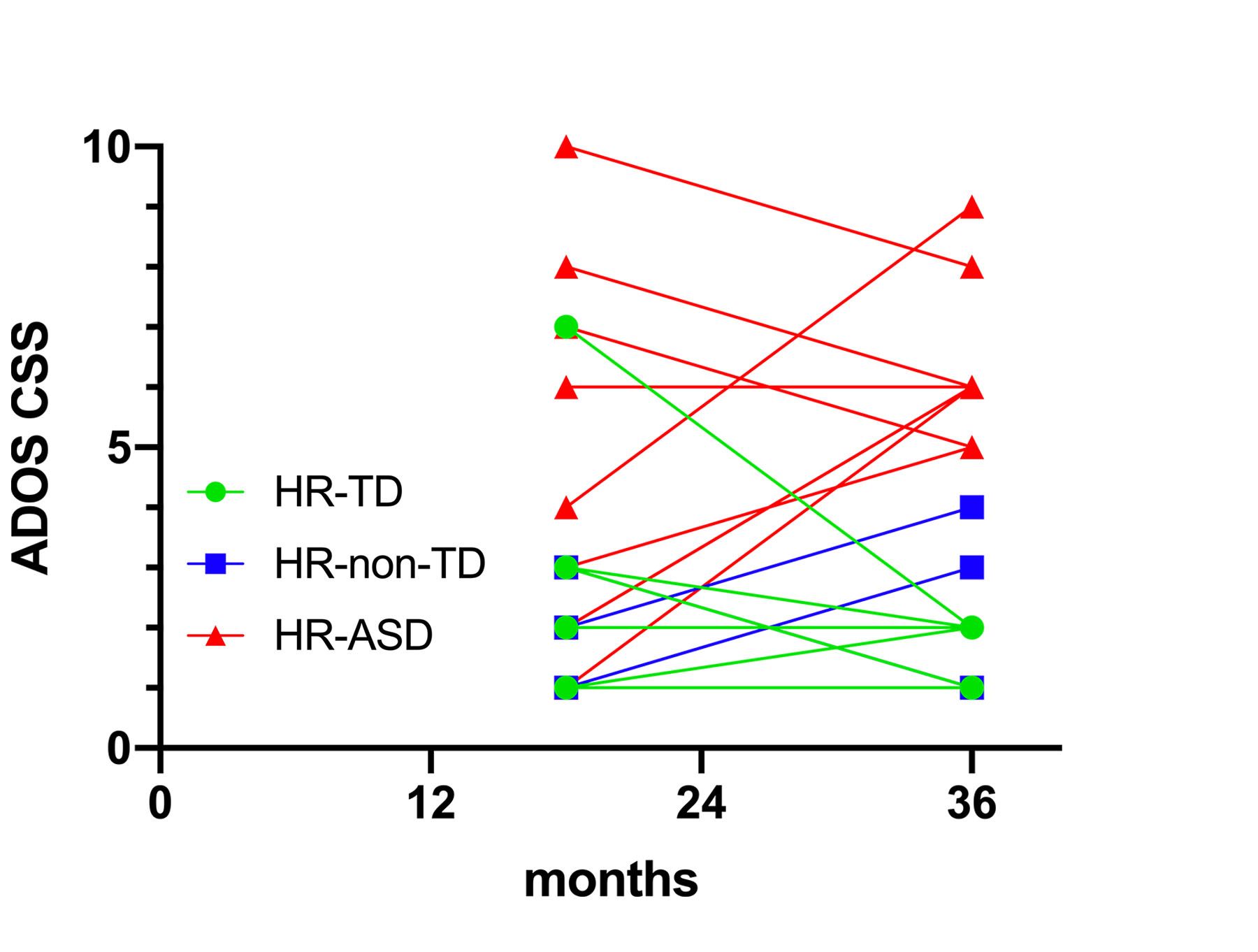

Supplement: Supplementary Figure 1 — Graphic representation of individual ADOS CSS at 18 and 24 months of age in our sample (n = 20 HR participants). Color code represents individual diagnosis outcome at age 3. HR-TD: high risk for ASD with typical development; HR-non-TD: high risk for ASD with atypical development; HR-ASD: high risk for ASD with ASD. [file Image_1.JPEG]

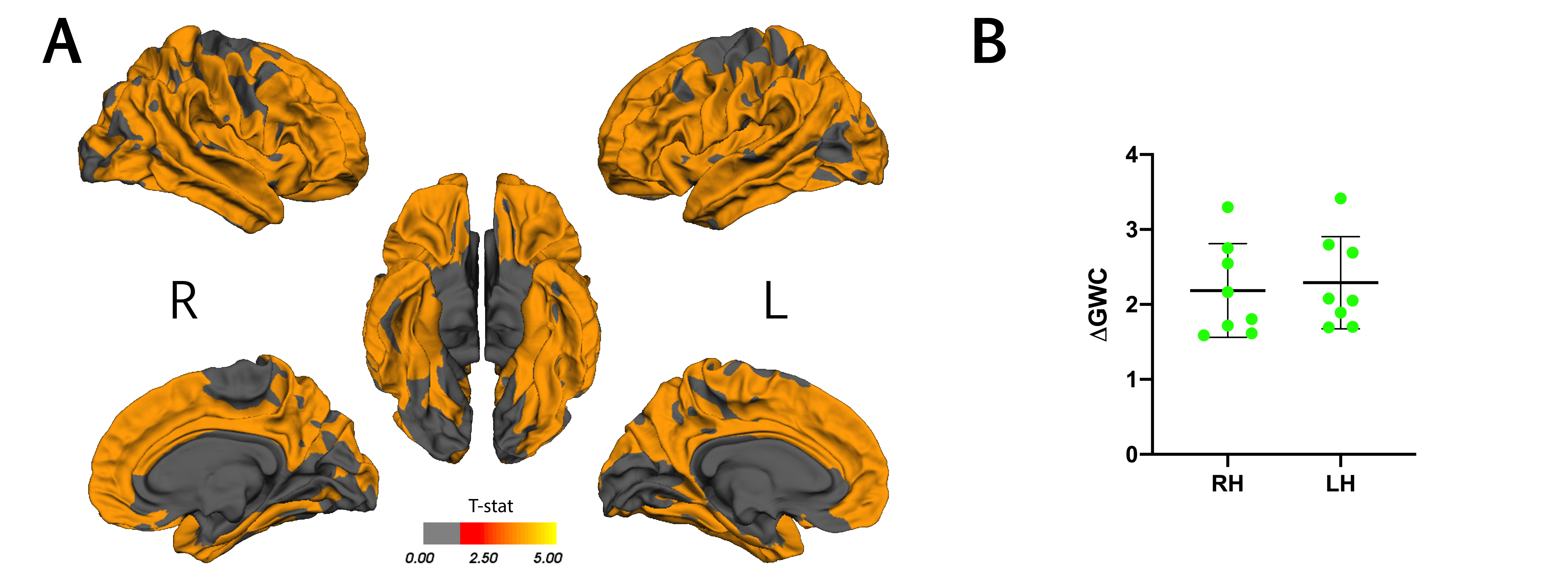

Supplement: Supplementary Figure 2 — (A) Clusters with a significant effect of time on gray-white matter contrast (GWC) in the HR-TD group. Clusters with cluster-wise P-value (CWP) < 0.05 only are displayed. Color code corresponds to P-value of the vertex with the maximal P-value (Pvm) of each cluster. (B) On the right are plotted for each hemisphere the individual GWC rates of change (ΔGWC) within each significant cluster (one per hemisphere). We found no significant difference between both clusters (paired t-test, p = 0.15). [file Image_2.JPEG]

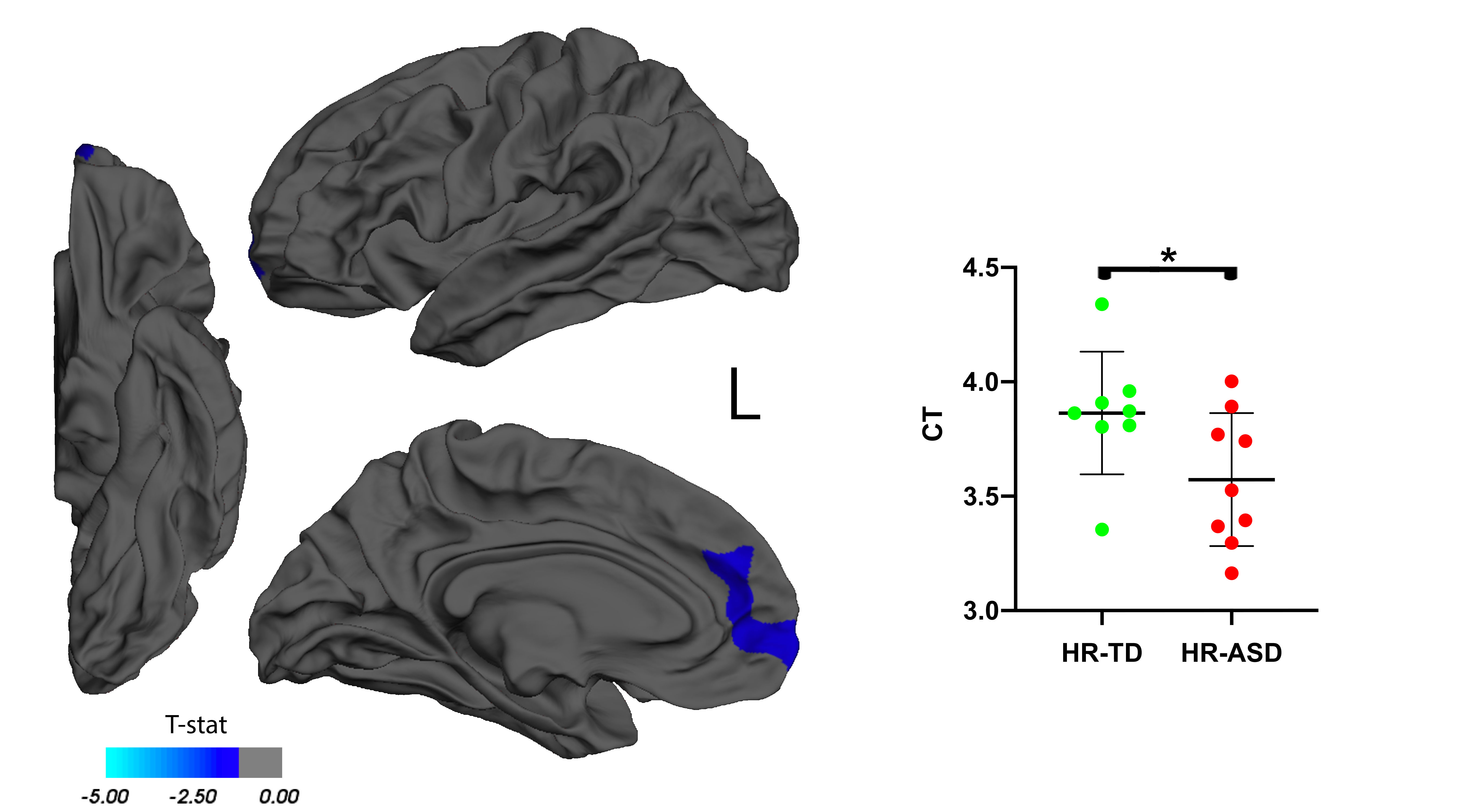

Supplement: Supplementary Figure 5 — Association between cortical thickness at age 12–24 months and diagnostic outcome at 36 months of age (HR-ASD or HR-TD). The single cluster with significantly smaller CT in HR-ASD compared to HR-TD (CWP < 0.05) is displayed. We found no significant cluster in the right hemisphere. Color code corresponds to P-value of the vertex with maximal P-value (Pvm) of the displayed cluster. On the right, individual CT-values are displayed in function of diagnosis outcome for the displayed cluster. ∗p < 0.05. CWP, cluster-wise P-value. [file Image_5.JPEG]

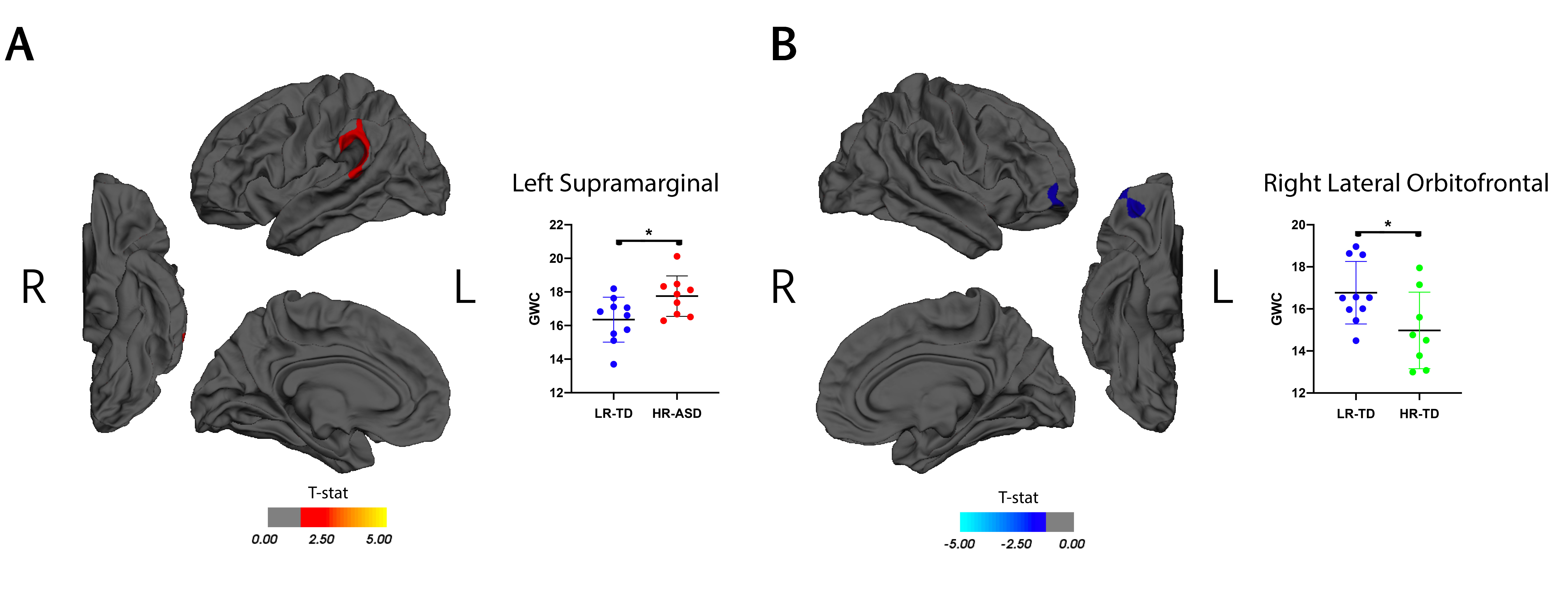

Supplement: Supplementary Figure 6 — Results of supplementary analysis comparing LR-TD with HR-ASD (A) and LR-TD with HR-TD (B). Color code corresponds to P-value of the vertex with maximal P-value (Pvm) of the displayed clusters. On the graphs, individual GWC values are displayed in function of diagnosis outcome for the displayed clusters. [file Image_6.JPEG]

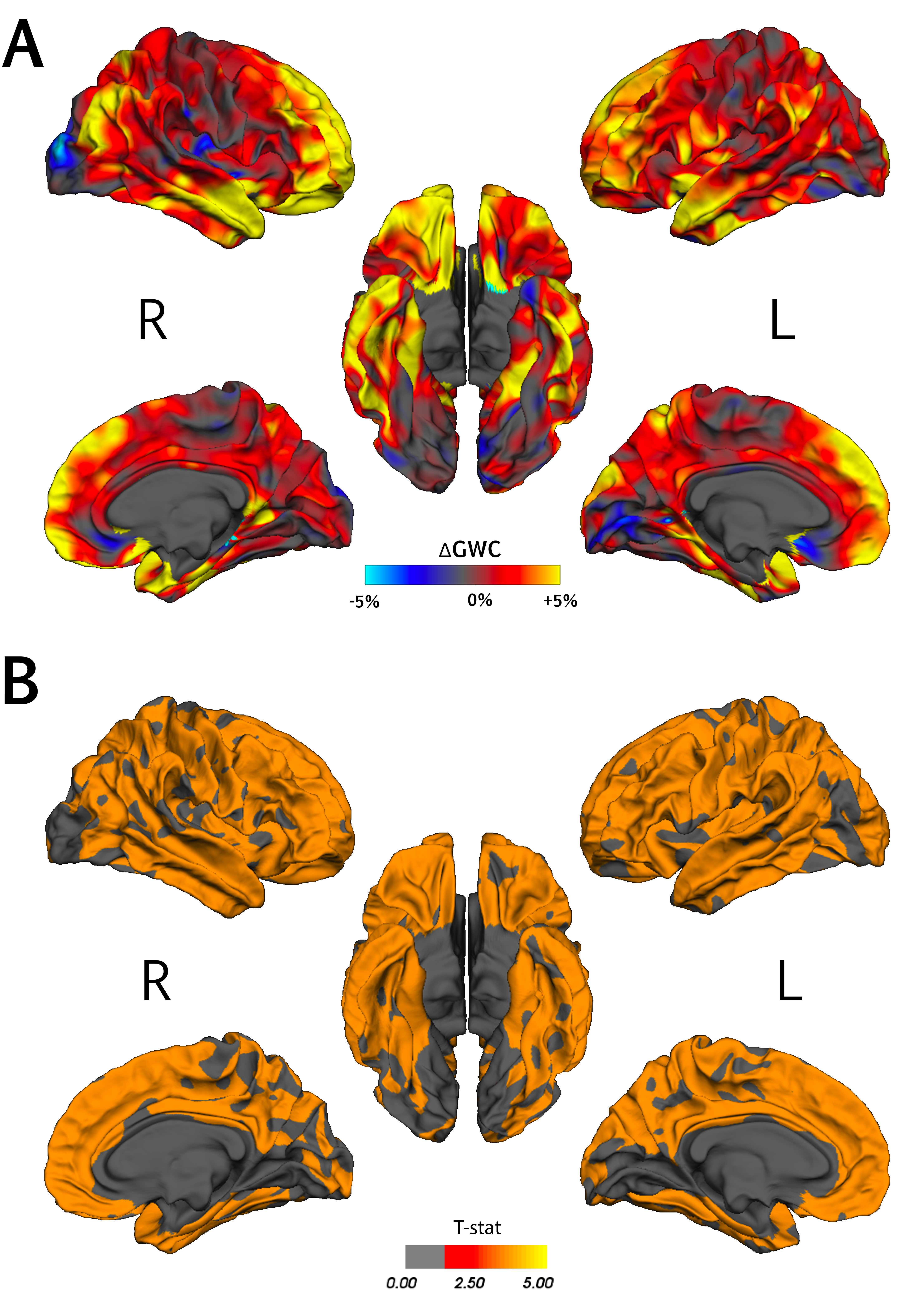

Supplement: Supplementary Figure 7 — (A) Effect of time on GWC within LR toddlers with typical development outcomes at 3 years (LR-TD) represented with vertex-wise ΔGWC values mapped on the common FreeSurfer template. (B) Clusters with a significant effect of time on gray-white matter contrast (GWC) in the LR-TD group. Clusters with cluster-wise P-value (CWP) < 0.05 only are displayed. Color code corresponds to P-value of the vertex with the maximal P-value (Pvm) of each cluster. [file Image_7.JPEG]
